# Transcatheter arterial embolization for severe blunt liver injury in hemodynamically unstable patients: a 15-year retrospective study

**DOI:** 10.1186/s13049-021-00881-7

**Published:** 2021-07-14

**Authors:** Satoshi Tamura, Takaaki Maruhashi, Fumie Kashimi, Yutaro Kurihara, Tomonari Masuda, Tasuku Hanajima, Yuichi Kataoka, Yasushi Asari

**Affiliations:** Department of Emergency and Critical Care Medicine, Kitasato University School of Medicine, 1-15-1, Kitasato, Minami-ku, Sagamihara, Kanagawa 252-0374 Japan

**Keywords:** Hepatic trauma, Liver injury, Blunt trauma, Non-operative management, Angioembolization, Transcatheter arterial embolization, Interventional radiology

## Abstract

**Background:**

Transcatheter arterial embolization (TAE) is the first-line nonsurgical treatment for severe blunt liver injury in patients, whereas operative management (OM) is recommended for hemodynamically unstable patients. This study investigated the comparative efficacy of TAE in hemodynamically unstable patients who responded to initial infusion therapy.

**Methods:**

This retrospective study enrolled patients with severe blunt liver injuries, which were of grades III–V according to the American Association for the Surgery of Trauma Organ Injury Scale (OIS). Patients who responded to initial infusion therapy underwent computed tomography to determine the treatment plan. A shock index > 1, despite undergoing initial infusion therapy, was defined as hemodynamic instability. We compared the clinical outcomes and mortality rates between patients who received OM and those who underwent TAE.

**Results:**

Sixty-two patients were included (eight and 54 who underwent OM and TAE, respectively; mean injury severity score, 26.6). The overall in-hospital mortality rate was 6% (13% OM vs. 6% TAE, *p* = 0.50), and the hemodynamic instability was 35% (88% OM vs. 28% TAE, *p* < 0.01). Hemodynamically unstable patients who underwent TAE had 7% in-hospital mortality and 7% clinical failure. Logistic regression analysis showed that the treatment choice was not a predictor of outcome, whereas hemodynamic instability was an independent predictor of intensive care unit stay ≥7 days (odds ratio [OR], 3.80; *p* = 0.05) and massive blood transfusion (OR, 7.25; *p* = 0.01); OIS grades IV–V were predictors of complications (OR, 6.61; *p* < 0.01).

**Conclusions:**

TAE in hemodynamically unstable patients who responded to initial infusion therapy to some extent has acceptable in-hospital mortality and clinical failure rates. Hemodynamic instability and OIS, but not treatment choice, affected the clinical outcomes.

## Background

Nonoperative management (NOM) of blunt liver injury through transcatheter arterial embolization (TAE) is reportedly associated with a success rate of 80–97% when used with advanced techniques in interventional radiology (IR) [1]. In addition, guidelines recommend TAE as the first-line therapy in hemodynamically stable patients with blunt liver injury [2]. However, there are recommendations (Level of Evidence I) for laparotomy in patients who are hemodynamically unstable, and NOM should not be selected for the management of patients with hemodynamic instability [3]. A small case series reported that TAE could be useful for hemodynamically unstable patients in facilities that could have quick and accurate application of the procedure [4]; however, to date, there are no comparative studies of TAE and operative management (OM) in hemodynamically unstable patients with liver injury.

Our institution has an IR-equipped emergency department, which facilitates a quick TAE; therefore, we attempted to perform TAE even in hemodynamically unstable patients with liver injury. This retrospective study covering a 15-year study period was conducted to determine whether TAE for severe blunt liver injury is associated with poorer prognosis in hemodynamically unstable patients. Moreover, we comparatively evaluated the differences in prognosis between TAE and OM.

## Methods

### Study design and methodology

This retrospective observational study reviewed the data of all patients with severe blunt liver injury (American Association for the Surgery of Trauma [AAST] grades III–V) who were treated at the Kitasato University Hospital Emergency and Critical Care Center between 2005 and 2019. We excluded patients with cardiac arrest on arrival. Regardless of age, all patients who received OM or TAE as initial treatment were included in this study. The OM and TAE groups included patients who underwent laparotomy for hemostasis and embolization for the treatment of severe blunt liver injury, respectively.

Our facility was the only regional trauma center serving a population of more than 1 million people, and our IR physicians were full-time staff at this center. Therefore, a 24-h IR facility was available at the study center.

### Clinical management and procedures

All trauma patients received initial infusion therapy that consisted of a rapid 1–2-L infusion of crystalloid, albumin, and a blood transfusion. Patients in shock without elevated blood pressure received OM and underwent damage control surgery. When the clinical condition of the patient responded to initial infusion therapy, the patient underwent computed tomography (CT). When an intestinal injury was detected, the patient was transferred to the operating room (Op room) for OM. In patients with an extravascular leak of contrast on CT, TAE was performed in the IR room. A shock index > 1, despite undergoing initial infusion therapy, was defined as hemodynamic instability. The shock index was defined by the ratio of the heart rate to the systolic blood pressure (SBP).

For embolization, the celiac artery was selected and accessed using a 5-Fr shepherd hook-type catheter (Hanaco Disposable Torque Catheter, Hanaco Medical Co., Saitama, Japan) or a 5-Fr cobra-type catheter (Torcon NB Advantage Catheter, Cook Japan, Tokyo, Japan). In pediatric patients, we used 4-Fr catheters. The hemostasis site was selected using a microcatheter, and a TAE was performed. In principle, we performed selective embolization; however, embolization from the right and left hepatic arteries or a more proximal site was acceptable when the patient was hemodynamically unstable. The embolic agents included gelatin sponge (through a pumping method) and coils or N-butyl cyanoacrylate, if there was an arterioportal shunt or coagulopathy, although the choice of the embolization material was determined at the IR physician’s discretion.

### Data collection

From electronic and paper medical records, we collected data on age, sex, mechanism of injury, vital signs at the time of visit, base excess, fibrinogen, Injury Severity Score (ISS), Trauma and Injury Severity Score (TRISS), time from arrival to CT, time from arrival to admission into Op room/IR room, and operation/TAE time. The AAST classification [5] was used to grade patients based on intraoperative findings or retrospective examination of CT images. The following outcomes were compared between the two study groups: in-hospital mortality, number of units of blood transfusion within 24 h of admission, massive transfusions (≥ 10 units of red blood cells), length of intensive care unit (ICU) stay, hospitalization duration, complications, and clinical failure. Clinical failure was defined as patient death because of hemorrhage within 24 h of undergoing OM (OM group) and switching from TAE to OM (TAE group) owing to hemostatic challenges. Complications included biloma, hepatic ischemia, pseudoaneurysm, gallbladder necrosis, arterioportal shunt, and rebleeding, detected on CT at 1 week after admission. Patients were discharged or transferred to the other hospital when their condition was stabilized, and the follow-up period was defined as the duration of their stay in our hospital.

### Statistical analysis

Statistical analyses were conducted using JMP® (SAS Institute Inc., Cary, NC, USA), utilizing Student’s t-test, the chi-square test, and the Wilcoxon rank sum test for comparisons between the OM and TAE groups and between the stable TAE and unstable TAE groups. Values of *p* ≤ 0.05 indicated statistical significance. Pairwise deletion was performed when cases with missing data were used. Logistic regression analysis was conducted concerning the outcome and incorporated variables with *p* < 0.10 and treatment choice as the variables on univariate analysis. Multivariate analysis was performed after organizing the cointegrated variables. The odds ratios (ORs) for each explanatory variable were calculated.

## Results

**Fig. 1:**
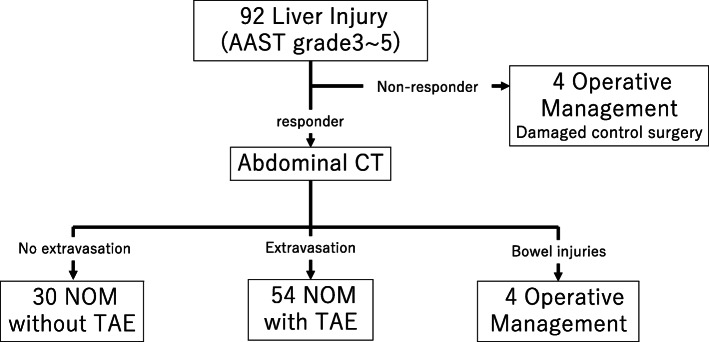
Management algorithm for patients with liver injury. NOM, nonoperative management; TAE, transcatheter arterial embolization

During the study period, 92 cases of severe blunt liver injury (AAST grade ≥ III) were admitted, of which 30 chose NOM without TAE for initial treatment; therefore, 62 cases (eight and 54 who underwent OM and TAE, respectively) were included in the analysis dataset. Four OM cases were treated with damage control surgery without undergoing CT because of no response to the initial infusion therapy (Fig. 1). The median age in this study population was 29.5 (interquartile range [IQR] 20–54) years. The mechanisms of injury were traffic accidents (*n* = 50, 81%), falls (*n* = 9, 14%), and compression trauma (*n* = 3, 5%); in this study population, the AAST grades were III (*n* = 36, 58%), IV (*n* = 21, 34%), and V (n = 5, 8%). The mean ISS was 26.6 ± 13.5. There were four (6%) deaths on admission, two (3%) clinical failures, 18 (29%) massive transfusions, and 34 (55%) complications in total. The median ICU stay of patients in this study population was 5.5 (IQR 3–12) days.

The comparison of the OM and TAE groups showed significant differences in the mechanism of injury (*p* = 0.02), blood pressure on arrival (OM 98.3 vs. TAE 125.9 mmHg, *p* = 0.01), Glasgow Coma Scale (GCS) score on arrival (OM 9.5 vs. TAE 15, *p* = 0.04), base excess (OM − 7.8 vs. TAE − 2.6, *p* < 0.01), ISS (OM 37.5 vs. TAE 24.9, p = 0.01), TRISS (OM 0.78 vs. TAE 0.96, *p* = 0.05), time from arrival to OM/TAE (OM 120.0 vs. TAE 76.1 min, p = 0.02), time for OM or TAE (OM 146.5 vs. TAE 29.4 min, p < 0.01), and hemodynamic instability (OM 88% vs. TAE 28%, p < 0.01) (Table 1). Outcomes had significant differences in ICU stay duration (OM 20.5 vs. TAE 5 days, p = 0.01) and massive transfusion (OM 75% vs. TAE 22%, *p* < 0.01). Clinical failures included one case each in the OM (death because of hemorrhage) and TAE (hypotension during IR that revealed portal vein injury, with good postoperative outcome) groups. Deaths in the TAE group were caused by cancer, cerebral infarction, and sepsis.

**Table 1:** Comparison between the operative management and transcatheter arterial embolization group

	OM(*n* = 8)	TAE(*n* = 54)	*P* value	All (*n* = 62)
Mean age (IQR)	35 (20–41)	28.5 (20–55)	1.00	29.5 (20–54)
Sex, (M/F)	7/1	42/12	0.51	49/13
Mechanism, n(%)			0.02	
trafic accident	4 (50%)	46 (85%)		50 (81%)
fall	4 (50%)	5 (9%)		9 (14%)
compression	0 (0%)	3 (6%)		3 (5%)
AAST grade of liber injury, n(%)			0.21	
gradeIII	3 (38%)	33 (61%)		36 (58%)
grade IV	3 (38%)	18 (33%)		21 (34%)
grade V	2 (25%)	3 (6%)		5 (8%)
sBP on arrival (mean)	98.3 ± 30.2	125.9 ± 26.7	0.01	122.7 ± 28.2
sBP on IR/Op room (mean)	106.4 ± 10.8	134.8 ± 3.9	0.02	131.5 ± 29.7
RR on arrival (meanl)	27.5 ± 4.4	25.2 ± 6.3	0.33	25.5 ± 6.1
GCS on arrival (median, IQR)	9.5 (6–15)	15 (14–15)	0.04	15 (13–15)
Base Excess on arrical (mean)	−7.8 ± 4.4	−2.6 ± 3.6	< 0.01	−3.1 ± 4.0
Fibrinogen on arrival (mean)	215.3 ± 105.1	106.9 ± 14.7	0.47	241.1 ± 106.3
ISS (mean)	37.5 ± 16.9	24.9 ± 12.3	0.01	26.6 ± 13.5
TRISS (median, IQR)	0.78 (0.21–0.97)	0.96 (0.86–0.99)	0.05	0.95 (0.85–0.98)
Time from Door to start OM/TAE	120.0 ± 109.4	76.1 ± 34.9	0.02	81.8 ± 51.7
Time for OM/TAE	146.5 ± 73.9	52.5 ± 5.2	< 0.01	65.0 ± 49.4
Hemodynamically unstable^a^	7 (88%)	15 (28%)	< 0.01	22 (35%)
In-hospital mortality	1 (13%)	3 (6%)	0.50	4 (6%)
Duration of ICU (median, IQR)	20.5 (10–35)	5 (3–9)	0.01	5.5 (3–12)
Length of stay (median, IQR)	62 (27–128)	23 (15–35)	0.03	26.5 (16–43)
Transfusion within first24h				
Units RBC (median, IQR)	11 (7–42)	0 (0–4.5)	< 0.01	0 (0–10)
Units FFP (median, IQR)	24 (10.5–43)	0 (0–10.5)	< 0.01	0 (0–14)
Units Plt (median, IQR)	0 (0–46)	0 (0–0)	0.10	0 (0–0)
Massive transfusion^b^	6 (75%)	12 (22%)	< 0.01	18 (29%)
Overall complication	4	30	0.45	34
Biloma	1 (13%)	12 (22%)	0.51	13 (21%)
Hepatic ischemia	0 (0%)	7 (13%)	0.15	7 (11%)
Psuedoaneurysm	0 (0%)	3 (6%)	0.36	3 (5%)
Gallbladder necrosis	1 (13%)	1 (2%)	0.19	2 (3%)
AP shunt	1 (13%)	5 (10%)	0.78	6 (10%
Rebleeding	0 (0%)	1 (2%)	0.60	1 (2%)
Clinical failure	1 (13%)	1 (2%)	0.19	2 (3%)

^a^Hemodynamically unstable: A shock index > 1, despite initial infusion therapy, was defined as hemodynamic instability

^b^Massive transfusion: ≥ 10 units of red blood cells

In the TAE group, we further compared the subgroups of patients who were unstable and stable and found significant differences (Table 2) in the AAST grade (*p* = 0.05), admission blood pressure (stable 133.0 vs. unstable 107.3 mmHg, *p* < 0.01), base excess (stable − 1.6 vs. unstable − 4.9, *p* < 0.01), ISS (stable 21.5 vs. unstable 33.9, p < 0.01), and TRISS (stable 0.98 vs. unstable 0.91, *p* = 0.02). Outcomes had significant differences in length of ICU stay (stable 4 days vs. unstable 8 days, *p* < 0.01) and massive transfusion (stable 10% vs. unstable 53%, *p* < 0.01).

**Table 2:** Comparison between unstable and stable patients who underwent transcatheter arterial embolization

	Stable (*n* = 39)	Unstable^a^ (*n* = 15)	*P* value
Mean age (IQR)	35 (20–56)	24 (17–32)	0.08
Sex, (M/F)	30/9	12/3	0.81
Mechanism, n(%)			0.34
trafic accident	34 (87%)	12 (80%)	
fall	4 (10%)	1 (7%)	
compression	1 (3%)	2 (13%)	
AAST grade of liber injury, n(%)			0.05
gradeIII	27 (69%)	6 (40%)	
grade IV	11 (28%)	7 (47%)	
grade V	1 (3%)	2 (13%)	
sBP on arrival (mean)	133.0 ± 3.9	107.3 ± 6.3	< 0.01
sBP on IR/Op room (mean)	143.6 ± 4.0	111.9 ± 6.4	< 0.01
RR on arrival (meanl)	24.8 ± 1.0	26.4 ± 1.6	0.4
GCS on arrival (median, IQR)	15 (14–15)	14 (9–15)	0.11
Base Excess on arrical (mean)	−1.6 ± 0.5	−4.9 ± 0.9	< 0.01
Fibrinogen on arrival (mean)	257.9 ± 16.9	209.1 ± 28.3	0.14
ISS (mean)	21.5 ± 1.8	33.9 ± 2.8	< 0.01
TRISS (median, IQR)	0.98 (0.9–0.99)	0.91 (0.78–0.98)	0.02
Time from Door to start TAE	44.1 ± 4.7	53.3 ± 7.8	0.32
Time for TAE	53.8 ± 4.7	48.7 ± 8.2	0.6
In-hospital mortality	2 (5%)	1 (7%)	0.83
Duration of ICU (median, IQR)	4 (3–7)	8 (5–21)	< 0.01
Length of stay (median, IQR)	21 (14–31)	42 (31–67)	< 0.01
Transfusion within first24h			
Units RBC (median, IQR)	0 (0–0)	10 (0–12)	< 0.01
Units FFP (median, IQR)	0 (0–6)	12 (2–21)	< 0.01
Units Plt (median, IQR)	0 (0–0)	0 (0–20)	0.19
Massive transfusion^b^	4 (10%)	8 (53%)	< 0.01
Overall complication	21	8	0.83
Biloma	7 (18%)	5 (33%)	0.24
Hepatic ischemia	7 (18%)	0 (0%)	0.03
Psuedoaneurysm	2 (5%)	1 (7%)	0.83
Gallbladder necrosis	0 (0%)	1 (7%)	0.11
AP shunt	5 (13%)	0 (0%)	0.06
Rebleeding	0 (0%)	1 (7%)	0.11
Clinical failure	0 (0%)	1 (7%)	0.11

^a^Unstable: A shock index > 1, despite initial infusion therapy, was defined as hemodynamic instability

^b^Massive transfusion: ≥ 10 units of red blood cells

On univariate analyses of outcomes, the GCS score (OR, 0.65; *p* < 0.01) and age (OR, 1.05; p = 0.05) were significant factors for in-hospital mortality (Table 3). However, multivariate analysis with GCS score, age, and TAE as objective variables showed that the GCS score (OR, 0.48; *p* < 0.01) and age (OR, 1.08; *p* = 0.04) were significant factors accounting for multicollinearity (Table 4).

**Table 3:** univariate analyses of outcomes for intergroup comparison

	In-hospital mortality		Duration of ICU≧7 days		Massive transfusion^b^		Complication	
	Odds ratio(95%CI)	p univariate analysis	Odds ratio(95%CI)	p univariate analysis	Odds ratio(95%CI)	p univariate analysis	Odds ratio(95%CI)	p univariate analysis
Age	1.05 (0.89–1.01)	0.05	1.00 (0.79–1.02)	0.83	0.99 (0.98–1.04)	0.61	0.99 (0.98–1.04)	0.33
Sex female	1.28 (0.12–13.41)	0.84	1.99 (0.54–7.37)	0.29	0.90 (0.24–3.41)	0.88	0.59 (0.16–2.19)	0.42
Severe liver injury (gradeIV,V)	1.42 (0.19–10.77)	0.74	1.20 (0.43–3.32)	0.73	1.19 (0.39–3.66)	0.76	6.61 (2.14–20.40)	< 0.01
sBP (arrival)	1.04 (0.92–1.01)	0.09	0.99 (0.99–1.03)	0.19	0.97 (1.01–1.06)	< 0.01	0.99 (00.99–1.03)	0.22
RR (arrival)	0.91 (0.91–1.31)	0.33	1.06 (0.86–1.03)	0.16	1.05 (0.87–1.05)	0.32	1.00 (0.92–1.09)	0.98
GCS (arrival)	0.65 (1.13–2.09)	< 0.01	0.83 (0.99–1.47)	0.04	0.79 (1.05–1.54)	0.01	0.99 (0.85–1.20)	0.90
Base Excess (arrival)	0.97 (0.78–1.37)	0.81	0.77 (1.09–1.55)	< 0.01	0.84 (1.02–1.39)	0.02	1.00 (0.87–1.14)	0.99
Fibrinogen (arrival)	1.00 (0.99–1.01)	0.86	1.00 (0.998–1.01)	0.11	0.99 (1.00–1.02)	0.01	1.00 (0.99–1.00)	0.84
ISS	1.05 (0.90–1.02)	0.19	1.08 (0.88–0.98)	< 0.01	1.18 (0.77–0.93)	< 0.01	0.99 (0.97–1.05)	0.57
Time from Door to start OM/TAE	0.98 (0.98–1.07)	0.19	1.01 (0.98–1.00)	0.09	1.01 (0.98–1.00)	0.28	1.00 (0.99–1.01)	0.84
Time for OM/TAE	0.97 (0.97–1.10)	0.13	1.01 (0.98–1.00)	0.06	1.00 (0.98–1.01)	0.44	0.99 (0.99–1.02)	0.24
Hemodynamicaly unstable^a^	1.90 (0.25–14.52)	0.54	7.03 (2.19–22.59)	< 0.01	15.8 (4.08–60.74)	< 0.01	0.96 (0.33–2.78)	0.94
TAE	0.41 (0.04–4.53)	0.50	0.08 (0.00–0.73)	< 0.01	0.10 (0.02–0.53)	< 0.01	2.23 (0.41–12.05)	0.33

^a^Hemodynamically unstable: A shock index > 1, despite initial infusion therapy, was defined as hemodynamic instability

^b^Massive transfusion: ≥ 10 units of red blood cells

**Table 4:** multivariate analyses of outcomes for intergroup comparison

	In-hospital mortality		Duration of ICU≧7 days		Massive transfusion^b^		Complication	
	Odds ratio(95%CI)	p multivariate analysis	Odds ratio(95%CI)	p multivariate analysis	Odds ratio(95%CI)	p multivariate analysis	Odds ratio(95%CI)	p multivariate analysis
Age	1.08 (0.98–1.19)	0.04						
Severe liver injury (gradeIV,V)							8.43 (2.51–28.30)	< 0.01
GCS (arrival)	0.48 (0.25–0.93)	< 0.01	0.94 (0.72–1.21)	0.62	0.95 (0.73–1.22)	0.67		
Fibrinogen (arrival)					0.99 (1.00–1.02)	0.13		
Time from Door to start OM/TAE			1.00 (0.99–1.02)	0.32				
Hemodynamicaly unstable^a^			3.80 (0.99–14.58)	0.05	7.25 (1.58–33.39)	0.01		
TAE	0.11 (0.00–10.70)	0.42	0.25 (0.02–2.91)	0.23	0.23 (0.03–2.11)	0.2	4.60 (0.70–30.40)	0.09

^a^Hemodynamically unstable: A shock index > 1, despite initial infusion therapy, was defined as hemodynamic instability

^b^Massive transfusion: ≥ 10 units of red blood cells

As observed on univariate analysis, the GCS score (OR, 0.83; p = 0.04), base excess (OR, 0.83; p = 0.04), ISS (OR, 1.08; *p* < 0.01), hemodynamic instability (OR, 7.03; *p* < 0.01), and TAE (OR, 0.08; *p* < 0.01) were significant factors for length of ICU stay > 7 days (Table 3); however, in multivariate analysis adjusted for multicollinearity, only hemodynamic instability (OR, 3.80; *p* = 0.05) showed significant associations (Table 4).

Concerning massive transfusion, the SBP on arrival (OR 0.97, *p* < 0.01), GCS score (OR 0.79, *p* = 0.01), base excess (OR 0.84, *p* = 0.02), fibrinogen levels (OR, 0.99; p = 0.01), ISS (OR, 1.18; p < 0.01), hemodynamic instability (OR 15.8, *p* < 0.01), and TAE (OR, 0.10; *p* < 0.01) were significant factors on univariate analysis (Table 3). However, only hemodynamic instability (OR, 7.25; p = 0.01) showed significant associations on multivariate analysis, accounting for multicollinearity (Table 4). Only severe liver injury (grades IV and V; OR, 6.61; *p* < 0.01) showed significant associations for the development of complications on univariate and multivariate analyses (Tables 3 and 4).

## Discussion

The present study showed that in the treatment of severe blunt liver injury, the mortality rate was 6% in patients with hemodynamic instability who underwent TAE but responded to initial infusion therapy. TAE for hemodynamically unstable patients did not increase the mortality rate as compared with that for the stable group. A recent observational study from a trauma center reported a mortality rate of 3–8% for blunt liver injury and 15% for liver injuries of grades IV and V [6, 7], with comparable results. The choice of treatment was not a predictor of outcome; the GCS score on arrival was a predictor of in-hospital mortality, and hemodynamic instability was an independent predictor of length of ICU stay ≥7 days and massive blood transfusion. AAST grades IV and V were predictors of complications.

It has been reported that under certain conditions, TAE for hemodynamically unstable patients with liver injury does not increase the mortality rates. Previous studies have shown that factors contributing to failed NOM include high ISS, need for massive transfusion, hypotension on hospital arrival, high AAST, and intraperitoneal contrast extravasation [8–10]; however, some controversy prevails because AAST has been reported to be unrelated to the NOM failure rate [11–13].

A cohort study of 3627 patients with severe blunt liver injury of AAST Grade IV or higher reported that SBP < 90 mmHg was more likely to result in failed NOM (OR, 2.07) and that higher rates of NOM failure and mortality in hypotensive patients were associated with higher rates of NOM [13].

There are a few case reports of successful NOM with TAE for hemodynamically unstable patients [14–16]; however, a recent observational study reported that failure and mortality from NOM with TAE were independent of the hemodynamic status, with hemodynamic instability being defined as a case where the patient required rapid infusion or transfusion to maintain a SBP > 90 mmHg [4].

The success rate of TAE in patients with cardiovascular instability may depend on how quickly the procedure is initiated and completed. A historical cohort study [17] at the same institution reported that the introduction of a protocol wherein CT and TAE were performed within 30 min in cases of a response to the initial infusion therapy, even in cases where the patient was in shock at the time of admission, resulted in a decreased rate of OM without alterations in the failure or mortality rate. However, it was reported that only 6% of the NOM patients underwent TAE in facilities with IRs situated far from the trauma unit [18]. In this study, good access to IR and shortening of the duration from ER visit to TAE could have contributed to the results.

Patients who received TAE had fewer massive transfusions and shorter ICU stays than those who received OM. These results are consistent with those from previous reports [6, 7] and suggested that TAE is less invasive than OM and thus, results in fewer transfusions and a faster recovery. However, in multivariate analysis, the hemodynamic status was the only predictor of ICU stay and massive transfusion, and may not depend on treatment. Regarding the complications, only AAST showed a correlation. This finding was consistent with reports, in which major complications after NOM occurred only in patients with AAST grade III or higher injuries [12]. Moreover, our findings showed that risk factors for complications in 453 NOM cases were AAST grade IV (OR, 4.4) and V (OR, 12) injuries, independent of other factors [19]. The most common complications after TAE are hepatic necrosis, abscess, and biloma, according to a systematic review [20]. Complications are reported to occur in 70% of cases [21], suggesting that TAE may increase the complication rate [22]. There are reports that embolization should be undertaken more selectively than at the level of the proper hepatic artery to reduce complications [23, 24]. In the present study, we attempted to use selective embolization when the circulation dynamics permitted it.

There were more severe cases in the OM than in the TAE group because of the inability to perform CT when the patient was hemodynamically unstable and did not respond to initial infusion therapy. The mortality rate in severe blunt liver injury requiring OM is > 50% [25], and there are two ways to effectively utilize TAE in such cases. The first option is through resuscitative endovascular balloon occlusion of the aorta (REBOA), which has been reported to improve prognosis in severe trauma refractory to initial infusion therapy [26]. Thus, the inclusion of REBOA in our strategy may have further improved the prognosis in the TAE group. The other option is to effectively utilize TAE in a hybrid ER, where all examinations and treatments for trauma have been performed in a single station composed of a carbon-fiber fluoroscopic table with a self-propelled C-arm combined with a sliding gantry CT scanner. The hybrid ER has been reported to increase the rate of IVR, shorten the time to treatment initiation, and improve the prognosis following the treatment for severe trauma [27], and it is also effective in shortening the time to treatment because TAE can be conducted when CT is completed.

The limitations of this study include its single-center, retrospective design. Moreover, the findings of this study may not be easily generalizable as the study center was a facility, which has immediate access to TAE. This study included a wide range of unstable patients, and those with severe hemodynamic instability that was nonresponsive to initial infusion therapy underwent surgery. Therefore, the number of patients with OM was small and could not be simply compared to that of patients with TAE, which vary greatly in severity. Future prospective studies are needed to specifically control the institutional and patient enrollment criteria for the validation of the preliminary findings from this research. In addition, the long-term prognosis was not considered in this study.

## Conclusion

In centers with good access to TAE facilities, TAE could be an effective NOM option for hemodynamically unstable patients with severe blunt liver injury. Prospective and large-scale studies are needed to verify the specific criteria for treatment selection and for the application of these research findings in the clinical setting.

## Data Availability

The datasets generated and analyzed during the current study are not publicly available because of protection of personal information but are available from the corresponding author on reasonable request.

## Abbreviations

AAST: American Association for the Surgery of Trauma
CT: Computed tomography
GCS: Glasgow Coma Scale
ICU: Intensive care unit
IQR: Interquartile range
IR: Interventional radiology
ISS: Injury Severity Score
NOM: Nonoperative management
OM: Operative management
Op room: Operating room
OR: Odds ratio
REBOA: Resuscitative endovascular balloon occlusion of the aorta
SBP: Systolic blood pressure
TAE: Transcatheter arterial embolization
TRISS: Trauma and Injury Severity Score
